# Maternal allergic disease history affects childhood allergy development through impairment of neonatal regulatory T-cells

**DOI:** 10.1186/s12931-016-0430-8

**Published:** 2016-09-20

**Authors:** Shan-shan Meng, Rong Gao, Bing-di Yan, Jin Ren, Fei Wu, Peng Chen, Jie Zhang, Li-fang Wang, Yuan-ming Xiao, Jing Liu

**Affiliations:** 1 Department of Respiratory Medicine, The Second Hospital of Jilin University, No.218, Ziqiang Street, Nanguan District, Changchun, China; 2 Department of Critical Care Medicine, Zhongda Hospital, School of Medicine, Southeast University, Nanjing, China; 3 Department of Obstetrics and Gynecology, The Second Hospital of Jilin University, Changchun, China; 4 Department of Pediatrics, The Second Hospital of Jilin University, Changchun, China

**Keywords:** Allergy, Cord blood, Regulatory T-cell, Toll like receptor, Children

## Abstract

**Background:**

Maternal allergic disease history and impaired regulatory T-cells (Tregs) are critical risk factors for allergy development in children. However, the mechanisms that underlie these risk factors remain poorly defined. Therefore, the aim of this study was to assess whether maternal allergies affect the Tregs of offspring and lead to allergy development in childhood.

**Methods:**

A total of 332 mothers of healthy newborns (234 from no allergic mothers, 98 from allergic mothers) were recruited to this study. Detailed questionnaires were administered yearly to determine the allergy status of the mothers and the newborns from birth to 3 years of age. Cord blood samples obtained at the time of birth were analysed for Treg counts, as well Treg activity, based on their response to Toll-like receptor (TLR) stimuli such as lipid A (LPA) and peptidoglycans (PPG). Surface markers, associated genes, suppressive capacity, and cytokine levels of Tregs were also measured. Possible correlations between Treg activity and maternal or neonate allergies were assessed. In addition, environmental microbial content and other known risk factors for allergies were measured.

**Results:**

Cord blood mononuclear cells (CBMCs) from offspring with allergic mothers showed fewer CD4^+^CD25^+^FOXP3^+^ T cells, lower expression levels of associated genes, and reduced cytokine production of interleukin (IL)-10 and interferon-γ (*P* < 0.05), especially via the PPG-TLR2 pathway. Suppression of effector T cells by Tregs from children of mothers with allergies was impaired, especially IL-13 production by Type 2 T helper (Th2) cells (*P* = 0.026). Children who developed allergies in the first 3 years of life had lower numbers of CD4^+^CD25^+^FOXP3^+^ T cells and reduced *FOXP3* expression and IL-10 production as newborns (*P* < 0.05). Maternal allergic background was identified as a risk factor for allergy development in the children (*Odds ratio (OR)* = 2.46, 95 % CI = 1.05–5.79); while declining Treg numbers, IL-10 production, and *FOXP3* expression in neonates (PPG and LPA stimulated) were identified as independent risk factors for allergic diseases in offspring at 3 years of age after adjusting for maternal allergic history and environmental factors (*P* < 0.05).

**Conclusion:**

Maternal allergy correlated with impaired Tregs in neonates, and this could enhance the susceptibility of offspring to allergic diseases in early childhood due to an imbalance of Th1 and Th2 cells.

## Background

The incidence of childhood allergic diseases has exhibited a marked increase in recent years, with the incidence in China being greater than 3.3 % [1]. A family history of allergic diseases, especially maternal allergies, is associated with a high likelihood of childhood allergy development [2, 3]. Some prospective cohort studies of Western countries have shown that susceptibility to allergic diseases in children may occur early in life [4–6], and during this time period regulatory T-cells (Tregs) could be involved. However, analogous follow-up studies in China have not been performed.

Because Tregs influence the early steps of allergy initiation [7], it is important to measure Treg cell abundance. New research has found that CD4^+^CD25^+^ FOXP3^+^ T cells were found to be excellent markers for Tregs [8]. Stable expression of FOXP3 has been observed in mature Tregs, and this expression profile was found to be highly specific for Tregs [9]. Other Treg specific genes include glucocorticoid-induced TNFR-related protein (GITR) and lymphocyte activation gene 3 (LAG-3) [10, 11]. Furthermore, interleukin (IL)-10 is a relatively specific cytokine of Tregs, and secretion of this cytokine may act as an additional marker for Tregs [12]. The suppressive capacity of Tregs is required to maintain the balance of T helper (Th) cells, including Th1, Th2, and Th17 cells. Unchecked proliferation of these Th cell populations has been shown to play a key role in the pathogenesis of allergic diseases. Toll-like-receptor (TLR)-induced innate immune responses affect the early differentiation of T cells. In particular, TLR2 and TLR4 are induced by peptidoglycans (PPG) and lipid A (LPA), respectively, and have been shown to be important mediators of T cell differentiation. Immune system stimulation may be critical to early immune modulation, and several studies have reported that young children growing up in environments rich in microbial stimuli have a lower risk of allergy [13, 14]. At the molecular level, activation of the TLR2/4 pathways has been shown to upregulate Tregs and induce cytokine secretion in both children and animal models with allergy development [13–15].

We hypothesize that the extent of the T cell response to TLR2/4 stimulation may be influenced by a maternal background of atopy. Therefore, the aim of the present study was to examine whether TLR2/4 stimulation influences immunity in addition to the activity of T cell subsets such as Tregs. Secretion of cytokines IFN-γ (Th1 cells), IL-13 (Th2 cells), and IL-17 (Th17 cells) were also examined. Furthermore, follow-up studies were conducted to assess whether maternal allergic status affected the susceptibility of developing allergic diseases in the first three years of childhood.

## Methods

### Study populations

A total of 332 mothers of healthy newborns in the city of Changchun, China were recruited to this study. Of these mothers, 234 had no allergies, while 98 had been diagnosed with allergies. We defined maternal allergy history as a diagnosis of asthma [16], allergic rhinitis [17], and/or allergic eczema [18] with at least one positive specific IgE (sIgE). Enrollment occurred from January 2012 through December 2012 with the approval of the ethics committee of the Second Hospital of Jilin University (2012–003). Pregnant mothers were approached at least one month before delivery for consent and they completed a detailed questionnaire that assessed maternal and infant data. In addition, cord blood was collected from each neonate (*n* = 332). The study inclusion criteria consisted of healthy neonates and mothers with uncomplicated pregnancies. Exclusion criteria were preterm deliveries, parents with autoimmune diseases, perinatal infections, maternal antibiotic or systemic glucocorticoid use in the last trimester, rural newborns, unhealthy newborns, or paternal allergic diseases.

Follow-up studies were conducted from 2012 to 2015 by questionnaire when the children were 12, 24, and 36 months old. Follow-up questionnaires were only completed for 273 children. The population characteristics that were examined for the children included: vaccination status, pet exposure, air conditioner exposure, breast feeding, diet, frequency of viral respiratory infections, medications, household income, parental education, and cohabitation with a smoker. Allergic wheezing (as a proxy for asthma in children ≤ 5 years old [19]), allergic rhinitis, and allergic eczema were diagnosed by paediatricians based on clinical manifestations, the presence of at least one sIgE+, and parent-described patient symptoms. The cohort belonged to a low economic status group (≤20,000 Renminbi income/year) based on available data from the Statistics Department (http://www.stats.gov.cn/) on the Per Capita Disposable Income (PCDI) for the Jilin Province collected from 2012–2015.

### Dust collection and analysis

To evaluate the presence of environmental allergens, dust samples were collected from the living rooms of the pregnant women one month before delivery. Dust samples were subsequently collected from the children’s bedrooms at 12 ± 1, 24 ± 1, and 36 ± 1 months post delivery. All dust samples were collected on pre-weighed glass fibre filters. Collection was performed using vacuum cleaners with sampling nozzles (ALK, Horsholm, Denmark) according to a standardized protocol with photo and video instructions. Collected samples were stored at −20 °C until extraction. Dust-containing filters were weighed and extracted in a volume of 5–40 ml, as determined by the net dust weight (<0.5 g, 5 ml; 0.5–1.0 g, 10 ml; 1.0–2.0 g, 20 ml; > 2.0 g, 40 ml). Endotoxin levels were measured with a kinetic chromogenic Limulus amebocyte lysate (LAL) test and glucan levels were detected in an inhibition enzyme immunoassay an inhibition EIA [20].

### IgE detection

Total IgE and sIgE were measured in peripheral blood serum samples collected from both mothers and children using the RAST method. Levels of sIgE in serum against six foods (cow’s milk, hen’s egg, hazelnut, carrot, peanut, and wheat flour) and 13 inhalant allergens (Dermatophagoides farinae; Dermatophagoides pteronyssius; cat fur; dog fur; horse fur; Alternaria species; mugwort, plantain, birch, alder, hazel, and rye pollen; and grass pollen mix) were assayed. A positive sIgE was defined as one or more positive reactions ≥0.35 IU/mL to a panel of 19 common allergens.

### Lymphocyte stimulation and cytokine production

Cord blood (20 ml) and peripheral blood (5–10 ml) samples were collected from healthy neonates within 24 h of birth. Cord blood mononuclear cells (CBMCs) were isolated from cord blood by density gradient centrifugation with Ficoll-Paque PLUS (GE Healthcare, Piscataway, NJ, USA) after dilution in PBS (Gibco, Carlsbad, CA, USA). Cells were washed in RPMI 1640 (Gibco) and diluted in 10 % human serum (Gibco) to a concentration of 5 × 10^6^ cells/ml. CBMCs were cultured for three days in the presence of 10 μg/ml PPG, 0.1 μg/ml LPA (Sigma-Aldrich, St. Louis, MO, USA), or without a stimulation factor. Concentrations of IL-10, IL-13, IL-17, and interferon-γ (IFN-γ) in the CBMC supernatants were measured with a Human Cytokine Multiplex Assay kit (Bio-Rad, Munich, Germany) with Luminex detection. The lower limits of detection for the assay (pg/mL) were: 0.9 (IL-10), 2.1 (IL-13), 0.2 (IL-17), and 1.3 (IFN-γ).

### Flow cytometry

CBMCs were analysed by flow cytometry (BD Biosciences, San Jose, CA, USA) after three days of cultivation. For surface staining, 2 μl of anti-human CD4-fluorescein isothiocyanate (FITC), 1 μl of CD25-RPE-Cy5, 1 μl of IgG1-FITC (DakoCytomation, Glostrup, Denmark), and 0.5 μl of IgG2a RPE-Cy5 (BD Biosciences) were added. For CD4/CD25/FOXP3 co-staining, 8 μl of anti-human CD4-FITC and 4 μl of anti-human CD25-RPE-Cy5 antibodies were added to 1 × 10^6^ cells in 100 ml PBS. Cells were then permeabilized and FOXP3-PE or the corresponding isotype control antibodies were added. Data were analysed with CellQuest software (BD Biosciences).

### Real-time quantitative RT-PCR

CBMCs were collected after three days of cultivation and total RNA was isolated immediately with the TriPure Isolation Reagent (Roche, Mannheim, Germany). RNA concentration was determined with a NanoDrop2000 (Thermo Scientific, Waltham, MA, USA), and samples were stored at −20 °C. Reverse transcriptase were performed (Invitrogen, Karlsruhe, Germany) for cDNA synthesis. Specific primer pairs were designed with Primer Express software (Vector NTI Advance10). Quantitative real-time PCR was performed with an iCycleriQ-multicolor Real-Time PCR Detection System. SYBR Green (Applied Biosystems, Darmstadt, Germany) was used to detect double-stranded PCR products. The C_T_ means the number of PCR cycles which are required for the fluorescence signal to exceed the detection threshold value. The threshold cycle (C_T_) of each target product is associated with the amplification plot of 18S. The level of mRNA from individual gene is described as gene expression. The fold difference in gene expression was calculated based on the difference in normalized C_T_ values between the stimulation and non-stimulation groups. The relative quantitative results showed changes of gene expressions in stimulated samples when compared with those in unstimulated ones.

### Functional analysis of tregs

CD3^−^ T cells were isolated (CD3^−^ Cell Isolation kit, MiltenyiBiotec, Köln, Germany) and irradiated as antigen-presenting cells. CD3^−^ T cell purity reached 98 %. CD4^+^CD25^+^ T cells were isolated from CBMCs by tandem positive selection (MiltenyiBiotec). CD4^+^CD25^−^ T cells were collected from the flow through during CD25 selection. The purity of both the CD4^+^CD25^−^ and CD4^+^CD25^+^ T cell populations was > 95 %. CD4^+^CD25^−^ T cells (effector T cells) were labelled with 5 μmol/L CFSE (CellTrace™ CFSE Cell Proliferation Kit, Invitrogen), and were subsequently incubated for three days with irradiated CD3^−^ cells in co-culture with or without CD4^+^CD25^+^ T cells (Tregs). The co-cultures were stimulated with phytohaemagglutinin (0.8 μg/ml). CFSE-stained CD4^+^CD25^−^ T cells were isolated by flow cytometry and ^3^H-thymidine incorporation was used to measure their proliferation. Concentrations of cytokines IL-13, IL-17, and IFN-γ were measured in the cell culture supernatants using a Human Cytokine Multiplex Assay Kit. The resulting values were compared between the effector T cells only group and the effector T cells plus Tregs group. The suppressive capacity of the Tregs on the effector T cells was further compared between the maternal allergy and non-allergy groups.

### Statistical analysis

Data were analyzed using SigmaStat1.0 software. Differences in cell counts, gene expression, suppressive function, and cytokine production for the Tregs present in the neonate cord blood samples were compared between maternal allergy and non-allergy groups, and between children’s allergy and non-allergy groups. Correlations between newborn Treg function and the development of childhood allergy were analysed. Metric data were evaluated by either a *t*-test or the Mann–Whitney rank test with or without normal distribution. Data are reported as the mean ± standard error of the mean (SEM) or the median with a 25–75 % interquartile range (IQR), depending on their distribution. Attributes data were analyzed with a *X*^*2*^ test or Fisher’s exact test. Multiple logistic regression was performed for the correlation analyses. Odds ratio (OR) values were reported when applicable. Statistical significance was defined as a *P-*value < 0.05.

## Results

### Cohort characteristics

Table 1 reports the available data for the 332 mother/neonate pairs of the present cohort (including 234 pairs in the maternal non-allergy group and 98 pairs in the maternal allergy group). Total maternal IgE levels were significantly higher in the maternal allergy group compared with the maternal non-allergy group (*P* = 0.005). Otherwise, there were no distinguishing characteristics between the mothers of the two groups, except there were four allergic asthmatic mothers who took low doses of inhaled glucocorticoids at gestational weeks 20–30.

**Table 1:** Characteristics of mothers and neonates

	Mothers without allergies (*n* = 234)	Mothers with allergies (*n* = 98)	*P*-value
Maternal age, y (IQR)	26 (23–32)	28 (22–35)	0.26
Body mass index (IQR)	23.15 (19.98–26.89)	22.04 (19.05–28.04)	0.23
Smoking status, n (%)			
Non smoking	225 (96.15)	97 (98.98)	0.29
Smoking during pregnancy	0 (0)	0 (0)	
Smoking until pregnancy	9 (3.85)	1 (1.02)	0.29
Maternal total IgE, UI/ml (IQR)	26.4 (9.42–57.9)	68.5 (42.5–222.4)	**0.005**
Maternal allergies [sIgE (+)], n (%)	0 (0)	98 (100)	
Asthma	0 (0)	23 (23.47)	
Allergic rhinitis	0 (0)	66 (67.35)	
Allergic eczema	0 (0)	30 (30.61)	
Endotoxins in maternal bedroom, EU/mg (IQR)	14.77 (4.20–55.18)	11.73 (2.64–50.14)	0.10
β (1,3)-glucans in maternal bedroom, μg/mg (IQR)	3.42 (1.04–9.57)	2.33 (0.67–5.88)	0.16
Vaginal delivery, n (%)	210 (89.74)	82 (83.67)	0.14
Male neonate gender, n (%)	120 (51.28)	51 (52.04)	0.90
Gestational age, weeks (SEM)	39.91 (1.20)	39.93 (1.18)	0.94
Birth weight, g (SEM)	3403.3 (407.37)	3610.56 (427.19)	0.45
Birth length, cm (IQR)	51.75 (51.36–52.14)	53 (52.48–53.52)	0.19
Apgar 5 min score > 8, n (%)	234 (100)	98 (100)	

Data in boldface are significant with *P ≤* 0.05. Data are reported as either the mean (SEM) or median (IQR) depending on the data distribution

A follow-up study was conducted for the 273 children for whom a completed follow-up questionnaire was completed at 0–3 years of age (Table 2). There were 59 children that were lost to follow-up due to a change in address or telephone number. While approximately half (53.52 %) of the allergic children had allergic mothers, only 30.69 % of the non-allergic children had allergic mothers. It was observed that the children with maternal allergy histories were more prone to allergic diseases (*P =* 0.001). The average concentration of endotoxin dust in the samples collected from the bedrooms of the allergic children was less than that in the bedroom samples for the non-allergic children (*P* = 0.05). However, there was no difference in β (1,3)-glucan exposure, diet, frequency of viral respiratory infections, household income, parental education, or cohabitation with a smoker between the children with and without allergies.

**Table 2:** Physical and environmental characteristics of children included in the follow-up study

	Children without allergies (*n* = 202)	Children with allergies (*n* = 71)	*P*-value
Male, n (%)	97 (48.02)	38 (53.52)	0.49
Body weight at 3 years of age, g (SEM)	15.1 (3.47)	16.7 (4.21)	0.14
Height at 3 years of age, cm (IQR)	97.6 (92.1–111.6)	97.4 (91.8–107.2)	0.58
Allergic diseases during 0–3 years of age, n (%)			
All allergies [sIgE (+)]	0 (0)	71 (100)	
Wheezing	0 (0)	19 (26.76)	
Allergic rhinitis	0 (0)	50 (70.42)	
Allergic eczema	0 (0)	47 (66.19)	
Maternal allergic history, n (%)	62 (30.69)	38 (53.52)	**0.001**
Average composition in dust from children’s bedrooms			
Endotoxin, EU/mg (IQR)	13.65 (3.51–45.78)	11.65 (2.78–39.44)	**0.05**
β (1,3)-glucans, μg/mg (IQR)	2.67 (0.54–7.45)	2.19 (0.51–5.56)	0.21
Pet exposure, n (%)	5 (2.47)	0 (0)	0.33
Air conditioner exposure, n (%)	22 (10.89)	9 (12.68)	0.54
Breast fed, n (%)	135 (66.83)	50 (70.42)	0.66
Respiratory virus infections, n (%)			
< 3 per year	139 (68.81)	47 (66.20)	0.44
3–5 per year	35 (17.33)	14 (19.72)	0.28
> 5 per year	28 (13.86)	10 (14.08)	0.54
Low household income, n (%)	92 (45.54)	26 (36.62)	0.21
Low parental education (middle school and lower), n (%)	35 (17.33)	12 (16.90)	1.0
Co-habitation with a smoker, n (%)	10 (4.95)	3 (4.23)	1.0

Data in boldface are significant with *P ≤* 0.05. Data were reported as either the mean (SEM) or median (IQR) depending on the data distribution

### Treg abundance and activity

**Fig. 1:**
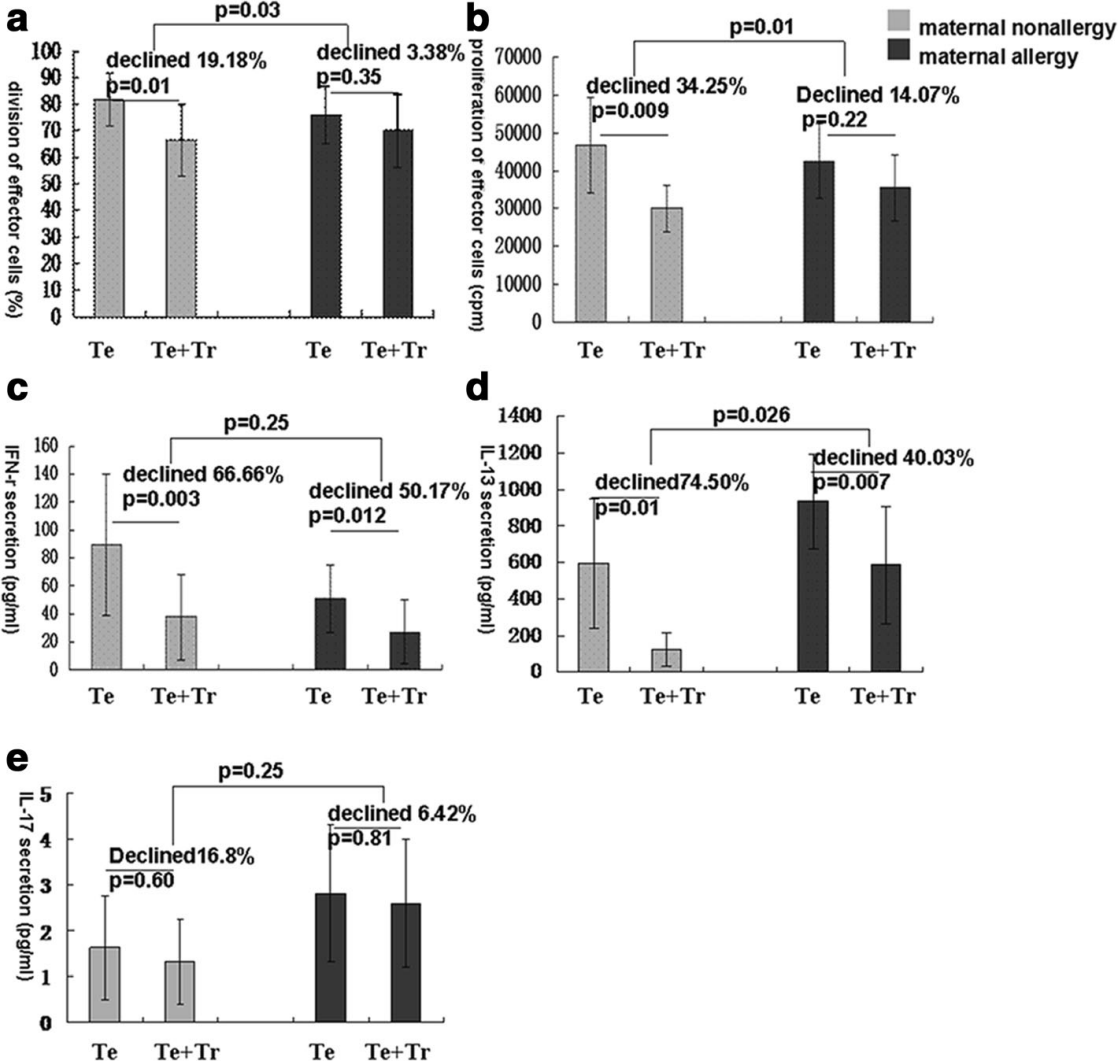
In vitro suppression of effector T cells by neonate Tregs. **a** Effector T cell division in the presence and absence of Tregs as determined by CFSE staining. **b** Effector T cell proliferation in the presence and absence of Tregs as determined by ^3^H-thymidine incorporation. **c**-**e** Effector T cell secretion of IFN-γ, IL-13, and IL-17 in the presence and absence of Tregs as measured by LUMINEX. Te: effector T cells; Tr: regulatory T cells (*n* = 8)

Treg abundance in the CBMCs (characterized as CD4^+^CD25^+^FOXP3^+^ T cells, Fig. 1) and cytokine production are listed in Table 3. Tregs were less abundant in the CBMCs collected from the neonates with maternal allergies than in those without allergies, especially following PPG stimulation (*P* = 0.01, Table 3). Both PPG/LPA-stimulated *FOXP3*, and PPG-stimulated *LAG3*, gene expression levels were lower in the neonates with maternal allergies than in the neonates from mothers without allergies (*P* < 0.05). Maternal allergic background also affected cytokine responses. IL-10 levels in the PPG-TLR2 innate immune pathway were lower in the offspring from the mothers with allergies than those from the mothers without allergies (*P* = 0.006), in agreement with the observations in CD4^+^CD25^+^FOXP3^+^T cells and the *FOXP3*/*LGA3* expression profiles for these groups. Production of the Th1 cytokine, IFN-γ, was also reduced upon LPA and PPG stimulation in the newborns with allergic mothers compared to the newborns with non-allergic mothers (*P* = 0.04 and *P* = 0.047, respectively), while increased production of PPG-stimulated IL-13 and IL-17 was observed as well (*P* = 0.021 and *P* = 0.049, respectively).

**Table 3:** Tregs and cytokines in CBMCs of offspring with and without maternal allergy

	Maternal non-allergy (*n* = 222)	Maternal allergy (*n* = 90)	*P*-value
CD4^+^CD25^+^ FOXP3^+^ T cells, % in CBMCs (SEM)			
U	1.78 (0.71)	1.57 (0.76)	0.12
LPA	2.05 (0.75)	1.81 (0.76)	0.08
PPG	2.33 (0.69)	1.99 (0.68)	**0.01**
FOXP3 gene expression, fold difference (IQR)			
LPA	3.14 (1.79–5.24)	2.50 (0.83–4.88)	**0.021**
PPG	3.36 (1.32–4.92)	2.69 (1.22–5.86)	**0.024**
GITR gene expression, fold difference (IQR)			
LPA	2.83 (1.53–6.24)	1.50 (0.95–3.03)	0.16
PPG	3.93 (1.87–8.46)	2.46 (0.95–5.89)	0.15
LAG3 gene expression, fold difference (IQR)			
LPA	2.83 (1.39–6.45)	1.53 (0.90–3.03)	0.12
PPG	2.93 (1.88–5.66)	2.00 (0.85–2.95)	**0.028**
IL-10 production, pg/ml (IQR)			
U	0.19 (0.01–0.27)	0.035 (0.01–0.23)	0.81
LPA	173.71 (109.24–198.26)	135.16 (91.37–241.89)	0.43
PPG	1104.47 (806.43–1519.56)	868.44 (603.76–1153.45)	**0.006**
IL-13 production, pg/ml (IQR)			
U	0.16 (0.01–0.33)	0.13 (0.01–0.36)	0.52
LPA	3.81 (1.45–7.80)	5.87 (1.48–13.21)	0.06
PPG	25.44 (12.41–53.98)	33.38 (13.14–61.53)	**0.021**
IFN-γ production, pg/ml (IQR)			
U	1.02 (0.01–1.99)	1.02 (0.45–1.99)	0.49
LPA	5.52 (3.47–17.64)	3.95 (1.64–9.66)	**0.04**
PPG	14.28 (6.57–33.42)	8.66 (5.02–18.96)	**0.047**
IL-17 production, pg/ml (IQR)			
U	0.09 (0.01–0.47)	0.12 (0.01–0.67)	0.38
LPA	2.39 (0.24–4.39)	2.26 (0.81–4.10)	0.86
PPG	2.7 (1.65–10.99)	4.03 (2.74–19.25)	**0.049**

*U* unstimulated, *LPA* stimulation with 0.1 μg/ml LPA, *PPG* stimulation with 10 μg/ml PPG

Data in boldface are significant with *P ≤* 0.05. Data are reported as the mean (SEM) or median (IQR) depending on the data distribution

In Table 4, Treg abundance, related gene expression, and cytokine production data are compared for neonate cord blood samples based on their childhood allergy status, independent of maternal allergy status. Children with allergies had a lower number of PPG-stimulated CD4^+^CD25^+^FOXP3^+^T cells (*P* = 0.01), reduced *FOXP3* expression in response to PPG and LPA (*P* = 0.02 and *P* = 0.01, respectively), and lower IL-10 production (*P* = 0.016), compared to the children without allergies.

**Table 4:** Tregs and cytokines in CBMCs of offspring with and without childhood allergies

	Children without allergies (*n* = 202)	Children with allergies (*n* = 71)	*P*-value
CD4^+^CD25^+^ FOXP3^+^ T cells, % in CBMCs (SEM)			
U	1.91 (0.67)	1.17 (0.61)	0.12
LPA	2.15 (0.73)	1.45 (0.54)	0.08
PPG	2.44 (0.63)	1.57 (0.49)	**0.01**
FOXP3 gene expression, fold difference (IQR)			
LPA	3.14 (1.60–5.25)	1.41 (0.70–2.64)	**0.01**
PPG	4.03 (1.32–6.02)	1.98 (1.25–4.14)	**0.02**
GITR gene expression, fold difference (IQR)			
LPA	2.14 (1.0–5.14)	2.79 (0.96–10.44)	0.12
PPG	2.42 (0.99–6.96)	3.67 (0.95–8.61)	0.44
LAG3 gene expression, fold difference (IQR)			
LPA	2.14 (0.61–4.65)	1.19 (0.68–3.36)	0.51
PPG	2.0 (0.54–3.73)	2.22 (0.63–4.74)	0.61
IL-10 production, pg/ml (IQR)			
U	0.10 (0.01–0.26)	0.15 (0.01–0.21)	0.35
LPA	177.93 (110.74–229.50)	135.28 (88.14–192.87)	0.09
PPG	1078.74 (730.84–1518.49)	830.74 (623.0–1038.23)	**0.016**
IL-13 production, pg/ml (IQR)			
U	0.16 (0.01–0.37)	0.21 (0.01–0.35)	0.63
LPA	4.5 (1.65–11.83)	5.07 (1.70–13.26)	0.80
PPG	31.70 (12.88–50.53)	30.59 (11.90–61.45)	0.53
IFN-γ production, pg/ml (IQR)			
U	1.02 (0.01–1.99)	1.66 (0.45–2.51)	0.09
LPA	5.08 (2.81–16.86)	4.91 (2.82–7.72)	0.55
PPG	13.15 (6.12–30.33)	10.22 (4.96–26.50)	0.53
IL-17 production, pg/ml (IQR)			
U	0.01 (0.01–0.47)	0.01 (0.01–0.43)	0.96
LPA	1.71 (0.19–4.39)	0.84 (0.01–2.80)	0.12
PPG	2.94 (1.28–11.41)	2.34 (0.74–6.48)	0.18

*U* unstimulated, *LPA* stimulation with 0.1 μg/ml LPA, *PPG* stimulation with 10 μg/ml PPG

Data in boldface are significant with *P ≤* 0.05. Data are reported as the mean (SEM) or median (IQR) depending on the data distribution

### The suppressive capacity of tregs

**Fig. 2:**
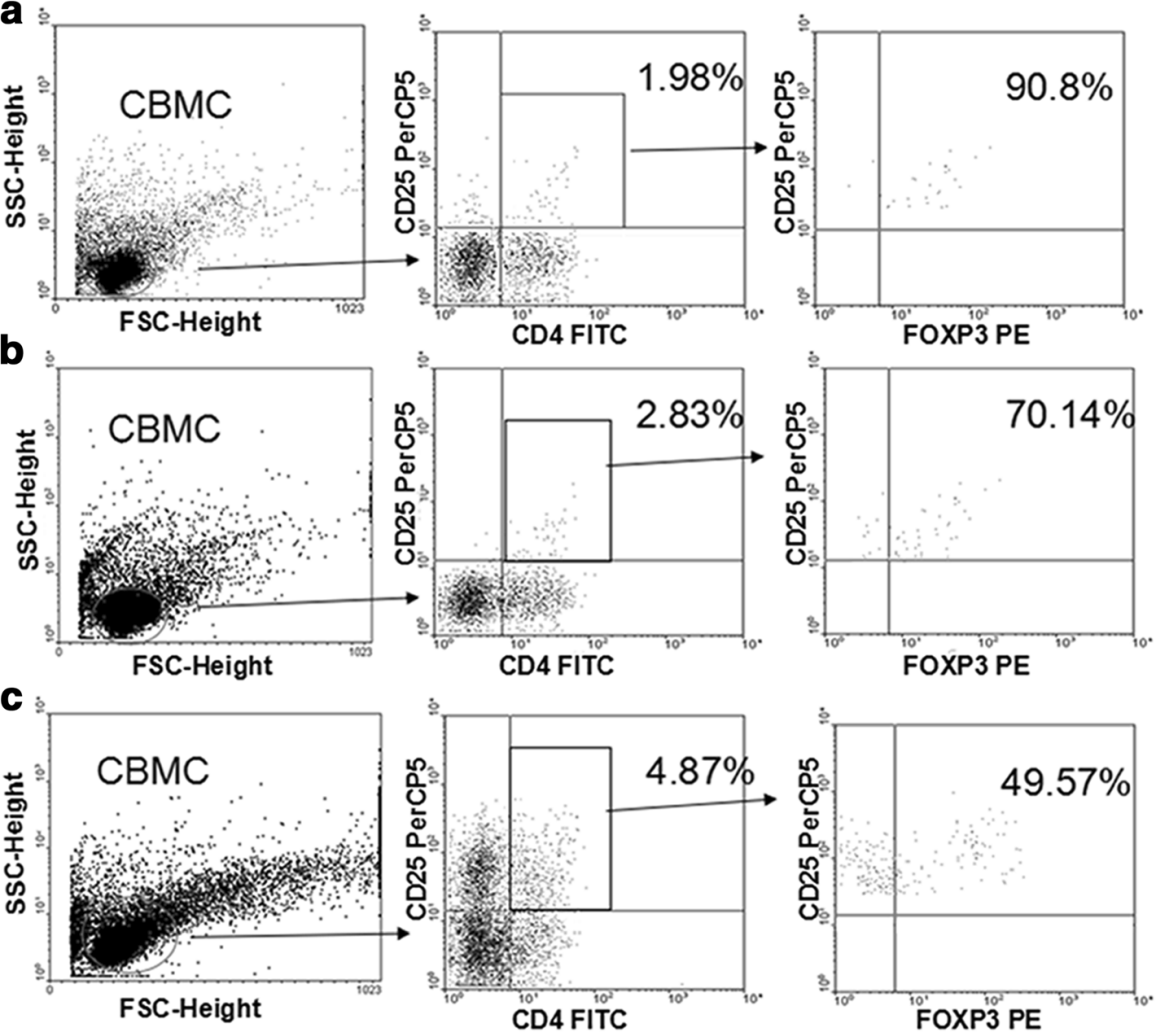
Percent of CD4^+^CD25^+^FOXP3^+^ T cells in untreated and stimulated CBMCs. CD4^+^CD25^+^FOXP3^+^ T cell populations were determined by fluorescent antibody staining and FACS. **a** Unstimulated CD4^+^CD25^+^FOXP3^+^T cells represented 1.80 % of the CBMC population (=90.8 % * 1.98 %). **b** CD4^+^CD25^+^FOXP3^+^T cells represented 1.98 % of the LPA-stimulated CBMC population (=70.14 % * 2.83 %). **c** CD4^+^CD25^+^FOXP3^+^T cells represented 2.41 % of the PPG-stimulated CBMC population (=49.57 % * 4.87 %). See Methods for details on CBMC culturing, stimulation, and fluorescence staining

Tregs from the children with allergic mothers suppressed the division and proliferation of effector T cells less efficiently than the Tregs from the children with non-allergic mothers (*P* = 0.03 and *P* = 0.01, respectively; Fig. 2). The Tregs of the former group also exhibited a significant decline in the production of IL-13 compared to the latter group (*P* = 0.026, Fig. 2). In contrast, production of IL-17 and IFN-γ were unaffected (*P* > 0.05, Fig. 2).

### Risk factors for allergic diseases

Maternal allergy was a significant risk factor for developing allergic diseases (*OR* = 2.46, *95 % CI* = 1.05–5.79; Table 5). After adjusting for maternal allergy, environmental exposure, diet, frequency of viral respiratory infections, household income, parental education, and co-habitation with a smoker as factors, the percent of CD4^+^CD25^+^FOXP3^+^T cells in the PPG-stimulated cord blood samples was found to negatively correlate with allergy status, allergic rhinitis, and allergic eczema in children, yet not with wheezing (Table 6). Conversely, a decrease in the percent of CD4^+^CD25^+^FOXP3^+^ T cells in the unstimulated cord blood samples was identified as a risk factor for wheezing (*adjusted OR* = 0.017, *95 % CI* = 0.001–0.46; Table 6). Decreased PPG-stimulated IL-10 production, as well as decreased LPA-stimulated and PPG-stimulated *FOXP3* expression, in cord blood were further identified as risk factors for a positive allergic status in children and allergic rhinitis (Tables 7 and 8, respectively).

**Table 5:** Association of allergy in children with maternal allergic history and endotoxin or β (1, 3)-glucan exposure

	OR (95 % CI) *P-*value		
	Maternal allergic history	Endotoxin (EU/mg)	β (1,3)-Glucans (μg/mg)
All allergies	**2.46 (1.05–5.79) 0.03**	**0.90 (0.75–1.02) 0.15**	1.05 (0.95–1.16) 0.27
Allergic wheezing	3.02 (0.68–13.35) 0.14	0.88 (0.75–1.03) 0.11	1.06 (0.92–1.20) 0.42
Allergic rhinitis	2.13 (0.815–5.56) 0.12	0.93 (0.85–1.01) 0.17	1.01 (0.95–1.07) 0.68
Allergic eczema	**3.21 (1.18–8.69) 0.02**	0.94 (0.84–1.06) 0.35	1.03 (0.93–1.15) 0.54

Data in boldface are significant with *P ≤* 0.05

**Table 6:** Association of allergy in children with % CD4^+^CD25^+^ FOXP3^+^ T in neonate CBMCs

	Adjusted OR (95 % CI) *P-*value		
	Unstimulated	LPA-stimulated	PPG-stimulated
All allergies	0.51 (0.18–1.51) 0.228	0.65 (0.23–1.85) 0.416	**0.12 (0.04–0.43) 0.01**
Allergic wheezing	**0.017 (0.001–0.46) 0.016**	2.15 (0.35–13.39) 0.41	0.36 (0.046–2.77) 0.32
Allergic rhinitis	0.49 (0.148–1.60) 0.23	0.39 (0.12–1.28) 0.12	**0.26 (0.074–0.91) 0.035**
Allergic eczema	1.08 (0.36–3.29) 0.88	0.57 (0.18–1.80) 0.34	**0.16 (0.044–0.56) 0.04**

Data in boldface are significant with *P* ≤ 0.05. Data were adjusted for maternal allergy, environmental and social factors

**Table 7:** Association of allergy in children with Treg-specific cytokine IL-10 levels

	Adjusted OR (95 % CI) *P-*value		
	Unstimulated	LPA-stimulated	PPG-stimulated
All allergies	0.17 (0.01–2.34) 0.19	0.65 (0.25–1.67) 0.37	**0.33 (0.13–0.86) 0.03**
Allergic wheezing	1.16 (0.07–18.98) 0.91	0.33 (0.07–1.51) 0.15	0.69 (0.15–3.26) 0.64
Allergic rhinitis	0.21 (0.07–5.94) 0.35	1.27 (0.41–3.98) 0.68	**0.29 (0.09–0.89) 0.04**
Allergic eczema	0.14 (0.04–4.97) 0.28	0.81 (0.27–2.43) 0.70	0.50 (0.17–1.46) 0.21

Data in boldface are significant with *P* ≤ 0.05. Data were adjusted for maternal allergy, environmental, and social factors

**Table 8:** Association of allergy in children with FOXP3 expression

	Adjusted OR (95 % CI) *P-*value	
	LPA-stimulated	PPG-stimulated
All allergies	0.78 (0.63–0.97) **0.04**	0.80 (0.65–0.97) **0.05**
Allergic wheezing	0.78 (0.52–1.16) 0.22	0.76 (0.52–1.13) 0.18
Allergic rhinitis	0.77 (0.59–0.99) **0.05**	0.85 (0.69–1.04) 0.12
Allergic eczema	0.83 (0.66–1.04) 0.11	0.81 (0.64–1.03) 0.08

Data in boldface are significant with *P* ≤ 0.05. Data were adjusted for maternal allergy, environmental, and social factors

## Discussion

In this birth cohort study, Tregs in the cord blood samples obtained from neonates with allergic mothers were fewer in number. The Tregs also exhibited compromised suppressive function, lower levels of expression for associated genes, and decreased IL-10 production. Moreover, the PPG-TLR2 innate immune response pathway was particularly affected. As a result, an increased susceptibility to allergies in early childhood (0–3 years old) was observed. Maternal allergic disease history and neonatal impairment of Tregs were both identified as risk factors for susceptibility to allergies in the children examined, independent of social and environmental factors for the children and their families. These results are consistent with those of previous studies of allergies in European populations [4, 5].

Many studies have reinforced the importance of in utero exposure for fetal immune development and the programming of susceptibility to asthma and allergic diseases [21]. In the present study, these factors were adequately considered to avoid potential interference. In addition, based on the results of a study by Schaub et al. where a higher percentage of allergic neonates with allergic mothers were more likely to have allergic fathers [5], paternal allergies were established as an exclusion criterion for the present study in order to focus on the effect of maternal allergic disease history on offspring. The study by Schaub et al. also reported increased birth weights and lengths for the newborns with allergic mothers [5]. However, no differences in birth weight or length between the groups of the present study were observed. This may be due to the similarity in the diets and medication use of the mothers included in the present cohort. Over the course of child development, many social and environmental factors should be considered when assessing influences on allergy development. Therefore, factors which have been shown to influence allergic disease development in children were considered in the present study, including: environmental exposure, diet, frequency of viral respiratory infections, household income, parental education, and co-habitation with a smoker [22]. It was observed that the endotoxin content in the living environments of the children with allergies was lower than in the living environments of the children without allergies. These results are consistent with those from Karvonen et al. where endotoxin exposure in early life was found to be closely related to the development of allergic diseases in children [23]. Consequently, microbial content was adjusted for in the analyses of risk factors for children’s allergic status.

As shown in Fig. 1a, b and c, FOXP3^+^ T cells represented > 90 % of the unstimulated CD4^+^CD25^+^ T cells. Therefore, CD4^+^CD25^+^ T cells were isolated from the unstimulated CBMCs to serve as a Treg population that would have its suppressive function examined in vitro. There were fewer CD4^+^CD25^+^FOXP3^+^ T cells present in both the neonates with allergic mothers and the neonates who developed allergies in early childhood. While the expression levels of GITR, LAG3, and FOXP3 were previously shown to positively correlate with each other [5], the specificity and sensitivity of their expression slightly differed in the TLR-mediated innate immune responses that were induced. It has been shown that Tregs secreting IL-10 play an important role in allergic diseases, independent of CD25^+^ Tregs [12]. Therefore, IL-10 production was assayed as a readout of function for this Treg subgroup. However, IL-10 is also secreted from Th2 cells. In the present data, PPG-stimulation reduced IL-10 secretion in the newborns with allergic mothers, while secretion of the Th2-specific cytokine, IL-13, increased (Table 3). These results suggest that IL-10 production is characteristic of this Treg subgroup in the TLR-mediated innate immune response. Overall, the risk factors for allergies in children of the present cohort included a reduced percentage of CD4^+^CD25^+^FOXP3^+^ T cells, reduced *FOXP3* expression, and PPG-stimulated IL-10 secretion (Tables 6, 7 and 8). However, the other cytokines assayed, IL-13, INF-γ, and IL-17, were not identified as risk factors (data not shown). To date, the traditional Th1/Th2 imbalance theory has not been sufficient to explain the complex pathogenesis for allergic diseases. In addition, Tregs, Th17 cells, Th9 cells, and other subgroups of T cells have all been implicated in allergic disease pathogenesis. Based on the importance of Tregs maintaining their suppressive function, we hypothesize that impairment of Tregs in neonates with allergic mothers leads to dysregulation of the differentiation of Th0 cells into Th1, Th2, and Th17 cells, and this is the driving force for allergy development in children.

When the children of this study were divided into those with or without allergies, regardless of maternal allergy status, down-regulation of Tregs according to the TLR2/4 pathway at the newborn stage partly predicted the children’s allergic status. However, it was not clear whether the down-regulation of Tregs derived exclusively from maternal allergy history. By using multiple logistic regression, exclusion of maternal allergic disease history and environmental factors led to the identification of a reduced number of CD4^+^CD25^+^FOXP3^+^ T cells, reduced *FOXP3* gene expression, and reduced IL-10 secretion in neonates as independent risk factors for the allergic status of children (Tables 6, 7, and 8).

It was previously demonstrated that impairment of Tregs in neonates with allergic mothers was more profound in the TLR2/4-mediated innate immune response pathways, due in part to genetic variations in the TLRs present in cord blood [24]. Genetic-immunological interactions of the TLR pathway influence Tregs early in life and are modulated by maternal allergic disease history. Moreover, it is likely these interactions are relevant for immune maturation in the development of allergic diseases in childhood. In addition to a genetic component, the time, frequency, and intensity of activation of TLR-mediated immune pathways are important for the maturation and differentiation of Tregs and other Th cells. For instance, increased microorganism exposure in early life in children and in mouse models has been shown to regulate the immune system by enhancing the Th1 pathway and weakening the Th2 pathway through TLR2/4 immune responses [25–27].

The results of the present study have revealed that maternal allergy status has a significant impact on Treg differentiation in the TLR2/4 immune pathway, yet not in the unstimulated pathway. The present results also confirm that the occurrence of allergic disease is the result of interactions between genetic and environmental factors. In particular, the neonatal period appears to be a key immune maturation period, and environmental endotoxin levels during this period can influence the extent of differentiation that takes place to generate different T cell subgroups in children via the TLR2/4 pathway. The number of Tregs present in fetal umbilical cord blood was observed to increase following PPG and LPA stimulation, thereby facilitating the differentiation of naive T cells into new Tregs. Maternal allergy history can influence modulation of differentiation and inhibit the differentiation rate of Tregs through the interactions of other T cell subgroups.

Thorburn et al. [28] showed that high fibre or acetate diets during pregnancy, which influence gut microbiota, can lead to marked suppression of allergic airway diseases in offspring by enhancing Treg numbers and function. These results are consistent with the observation that the intake of probiotics during pregnancy and early life have the potential to prevent childhood eczema [29] and IgE-associated allergies to some extent [30]. However, avoiding activation of the TLR-mediated immune pathway appears safer than a desensitization approach for preventing allergic disease development in the offspring of allergic pregnant women since the latter can up-regulate CD4^+^CD25^+^ T cells and IL-10 production in offspring [31, 32]. In the risk factor analysis performed in the present study, a decrease in PPG-stimulated Tregs was identified as a risk factor for children’s allergic status, allergic rhinitis, and allergic eczema. Meanwhile, a decrease in unstimulated Tregs was only a risk factor for allergic wheezing. Thus, micro-stimulation of the TLR2 and TLR4 pathways with adjustment of diet and environmental exposure may be an effective early intervention for preventing allergic disease in neonates that have lower numbers of Tregs or that have a maternal allergic disease history by establishing a more beneficial Th1/Treg balance.

## Conclusions

In summary, maternal allergic disease history and deficiency in Tregs in newborns are risk factors for the development of allergic diseases in children. The impairment of Tregs number and function in neonates could lead to susceptibility to allergic status and diseases through the imbalance of Th1/2 and Th17 cells. Because deficiency in Tregs is more obvious in the TLR2-mediated innate immune response pathway, we could seek possible micro-stimulation to up-regulate Tregs in the TLR2 pathway and provide early prevention for allergic diseases in children with maternal allergic histories.

## Abbreviations

CBMC: Cord blood mononuclear cell
C_T_: Threshold cycle
FITC: Fluorescein isothiocyanate
FOXP3: Forkhead/winged helix transcription factor
GITR: Glucocorticoid-induced TNFR-related protein
IFN-γ: Interferon-γ
IL: Interleukin
IQR: Interquartile range
LAG3: Lymphocyte activation gene 3
LAL: Limulus amebocyte lysate
LPA: Lipid A
OR: Odds ratio
PPG: Peptidoglycans
sIgE: Specific IgE
Th: T helper
TLR: Toll-like receptor
Treg: Regulatory T-cell
