# Erratum to: Maternal allergic disease history affects childhood allergy development through impairment of neonatal regulatory T-cells

**DOI:** 10.1186/s12931-016-0443-3

**Published:** 2016-10-21

**Authors:** Shan-shan Meng, Rong Gao, Bing-di Yan, Jin Ren, Fei Wu, Peng Chen, Jie Zhang, Li-fang Wang, Yuan-ming Xiao, Jing Liu

**Affiliations:** 1 Department of Respiratory Medicine, The Second Hospital of Jilin University, No.218, Ziqiang Street, Nanguan District Changchun, China; 2 Department of Critical Care Medicine, Zhongda Hospital, School of Medicine, Southeast University, Nanjing, China; 3 Department of Obstetrics and Gynecology, The Second Hospital of Jilin University, Changchun, China; 4 Department of Pediatrics, The Second Hospital of Jilin University, Changchun, China

*n.b. The errors and associated corrections described in this document concerning the original manuscript were accountable to the production department handling this manuscript, and thus are no fault of the authors of this paper. Additionally, the online manuscript has now been updated with these corrections accordingly.*

In the original publication of this article [1], figures 1 and 2 were inadvertently swapped. Figure 2 should therefore have been in the place of Figure 1 and vice versa. Please see below for the correct figure labels.

**Fig. 1:**
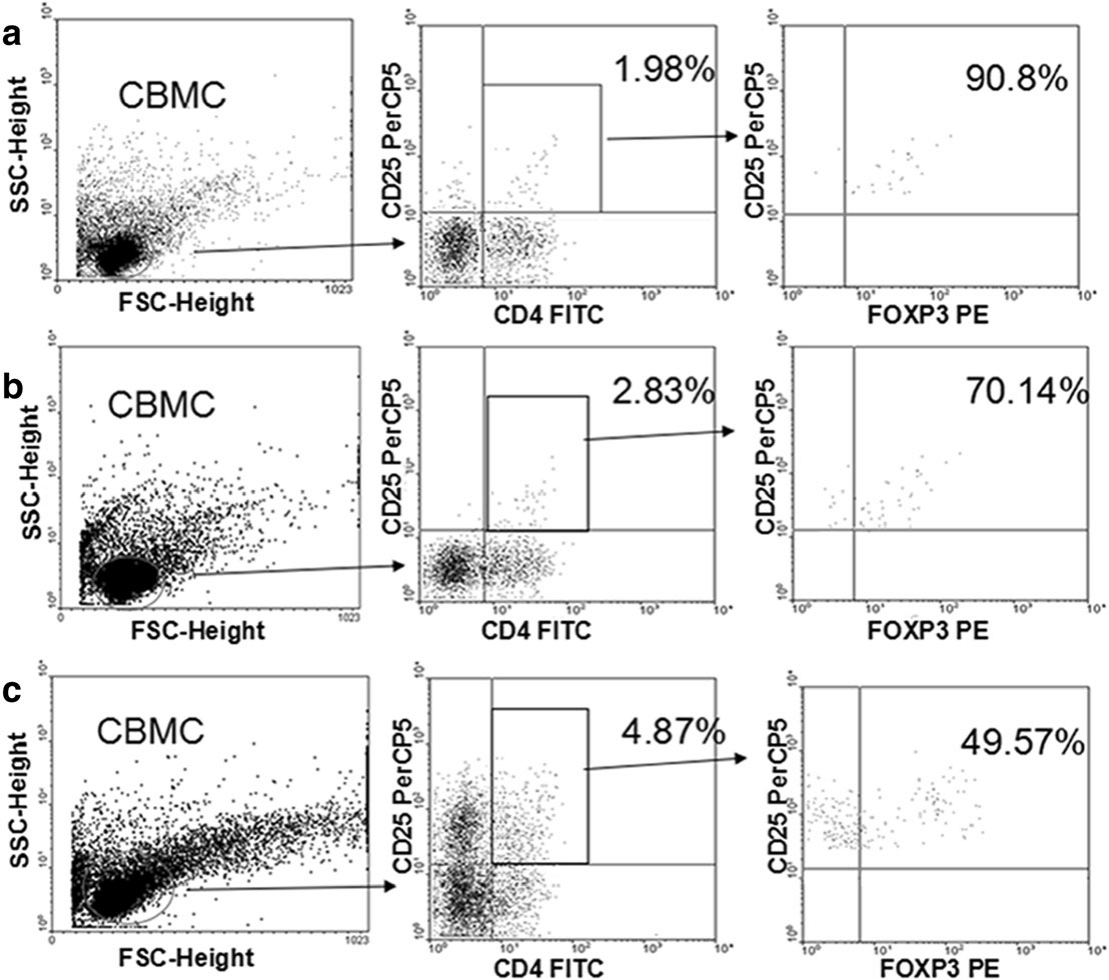
Percent of CD4^+^CD25^+^FOXP3^+^ T cells in untreated and stimulated CBMCs. CD4^+^CD25^+^FOXP3^+^ T cell populations were determined by fluorescent antibody staining and FACS. **a** Unstimulated CD4^+^CD25^+^FOXP3^+^T cells represented 1.80 % of the CBMC population (=90.8 % * 1.98 %). **b** CD4^+^CD25^+^FOXP3^+^T cells represented 1.98 % of the LPA-stimulated CBMC population (=70.14 % * 2.83 %). **c** CD4^+^CD25^+^FOXP3^+^T cells represented 2.41 % of the PPG-stimulated CBMC population (=49.57 % * 4.87 %). See Methods for details on CBMC culturing, stimulation, and fluorescence staining

**Fig. 2:**
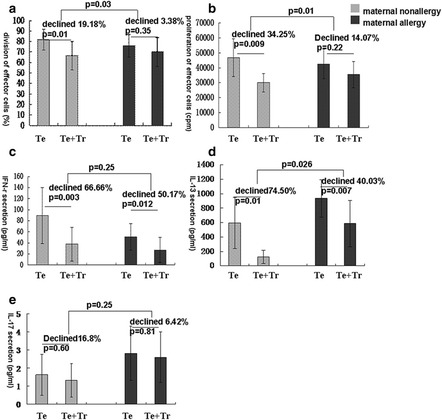
In vitro suppression of effector T cells by neonate Tregs. **a** Effector T cell division in the presence and absence of Tregs as determined by CFSE staining. **b** Effector T cell proliferation in the presence and absence of Tregs as determined by ^3^H-thymidine incorporation. **c**-**e** Effector T cell secretion of IFN-γ, IL-13, and IL-17 in the presence and absence of Tregs as measured by LUMINEX. Te: effector T cells; Tr: regulatory T cells (*n* = 8)
